# Study on naked eye tracing of inguinal sentinel lymph nodes in penile cancer patients with carbon nanoparticle suspension injection

**DOI:** 10.3389/fmed.2023.1139986

**Published:** 2023-03-09

**Authors:** Chengyi Liu, Pengcheng Xu, Song Shao, Mingshan Yang, Chao Li, Shuangjie Li, Wei Liu, Xiaobo Ding, Jici Ma, Guangyuan Li

**Affiliations:** ^1^Department of Urology, LU’AN Affiliated Hospital of Anhui Medical University, Lu'an, Anhui, China; ^2^Department of Orthopaedic, LU'AN Affiliated Hospital of Anhui Medical University, Lu'an, Anhui, China; ^3^Department of Urology, Shandong Cancer Hospital, Affiliated Tumor Hospital of Shandong First Medical University, Ji’nan, Shandong, China; ^4^The First Affiliated Hospital of Anhui Medical University, Anhui Public Health Clinical Center, Hefei, Anhui, China

**Keywords:** CNSI, penile carcinoma, sentinel lymph node, naked eye, tracer

## Abstract

**Objective:**

Exploratory study of the effect and clinical value of carbon nanoparticle suspension injection (CNSI) as a tracer for inguinal sentinel lymph nodes in penile cancer.

**Method:**

We selected 29 patients with penile cancer in our department from January 2019 to October 2022. According to whether the CNSI tracer was injected during the pathological biopsy of the inguinal lymph nodes, the enrolled patients were assigned to the control group, the group in which CNSI was injected 12 h before the surgery (12HBS group) and the group in which CNSI was injected 0.5 h before the surgery (0.5HBS group). Evaluating the effectiveness of CNSI as a lymphatic tracer involves analyzing the following: its safety, the statistical analysis of the detection rate (DR) of different groups, the number of lymph nodes sent for each case (NOLNSFEC), the difference of positive rate of lymphatic metastasis (PROLM), and operation time (OT).

**Results:**

The lymph nodes in the 12HBS group and 0.5HBS group had an obvious black staining appearance, and no adverse reactions or surgical complications were found. Most of the black-stained areas caused by CNSI injection were removed with penile excision, which did not affect the postoperative appearance. This did not affect the pathological analysis. The DR of lymph nodes in the 12HBS group was higher (*p* < 0.05) than that in the control group. More lymph nodes were removed for examination (*p* < 0.05), which improved the efficiency of surgery. Compared with the 12HBS group, the number of lymph nodes removed in the 0.5HBS group decreased (*p* < 0.05). The OT was shortened (*p* < 0.05), but there was no significant difference in the DR and PROLM.

**Conclusion:**

CNSI was applied to the naked-eye tracing of inguinal sentinel lymph nodes in penile cancer, which is safe and efficient. Injection of CNSI 0.5 h before surgery can help identify the “foremost position” of sentinel lymph nodes and reduce surgical trauma.

## Introduction

Lymph node metastasis is an important indicator of poor prognosis in penile cancer. The 5-year survival rate of patients without regional lymph node metastasis can reach 95 to 100%. The 5-year survival rate drops to 80% in the presence of a single inguinal lymph node metastasis. In the presence of multiple inguinal lymph node metastases, the 5-year survival rate drops to 50%; for pelvic and surrounding lymph node metastasis, it drops to 0% (1). However, at present, it is controversial whether to perform prophylactic lymphadenectomy in patients without inguinal lymphadenopathy on clinical examination. On the one hand, occult lymph node metastases are present in 10%–25% of patients with unpalpable lymphadenopathy in the groin (2–4). This means that 80% of patients who undergo inguinal lymph node dissection are overtreated and may suffer associated postoperative complications. On the other hand, the likelihood of lymphatic involvement is very high in patients with palpable inguinal lymph nodes, yet 30% to 50% of patients still have evidence of inflammatory lymphadenopathy rather than metastasis (5). Therefore, sentinel lymph node biopsy technology has been applied to the diagnosis and treatment of penile cancer. Since sentinel lymph nodes are not necessarily located in specific anatomical regions, in recent years, dynamic sentinel lymph node biopsy using tracers in primary lesions can not only detect hidden lymph node metastasis but also avoid unnecessary lymph node dissection.

At present, lymph node tracers mainly use methylene blue, colloid labeled with radioisotope 99Tcm, or indocyanine green alone or in combination (6–9). However, methylene blue metabolizes quickly; the staining duration is short; and the staining effect is often poor. Meanwhile, Radionuclide localization method needs to detect radionuclides emitted by low-energy gamma photons with the help of Handheld gamma probe and gamma camera to guide lymph node dissection (8), which is not only complicated to operate, but also has potential radioactive risks. In addition, indocyanine green tracer also requires Fluorescence imaging equipment to visualize lymph nodes (6, 10, 11). To overcome the abovementioned shortcomings, the carbon nanoparticle suspension injection (CNSI) selected in this project is used as a lymphatic tracer to achieve naked eye lymphatic tracing with exact effects and high safety.

## Patients and methods

### Data source and patient selection

A total of 29 patients with penile squamous cell carcinoma of stage ≥ pT1G2 and cN0 were selected in our hospital from January 2019 to October 2022. Preoperative CT scans of the chest, abdomen, and pelvis showed no distant metastases or pelvic lymphadenopathy. Regardless of palpable inguinal lymphadenopathy, all patients underwent partial phallectomy or total phallectomy plus urethroperineostomy. At the same time, a pathological biopsy of bilateral inguinal sentinel lymph nodes was performed. According to whether the CNSI tracer was injected during lymph node biopsy, the enrolled patients were divided into a control group, a group in which CNSI was injected 12 h before the surgery (12HBS group), and a group in which CNSI was injected 0.5 h before the surgery (0.5HBS group). Descriptive characteristics of these patients were shown in Table 1. The research protocol was approved by the Medical Ethics Committee of our hospital (approval number: 2021LL014), and all patients provided written informed consent.

**Table 1:** Descriptive characteristics of the entire cohort of 29 patients with ≥pT1G2 and cN0 penile squamous cell carcinoma.

Variables	Overall(%)
Age at surgery (yr), median(IQR)	69 (47–77)
**Clinical T stage**	
T1	8 (26)
T2	15 (52)
T3	2 (8)
Unknown	4 (14)
**Clinical N stage**	
N0	11 (38)
N1	18 (62)
Penis surgery method	
Partial penile resection	27 (93)
Total penile incision plus urethroperineostomy	2 (7)
Group	
Control group	17 (58)
12HBO group	6 (21)
0.5HBO group	6 (21)

### Materials and methods

#### CNSI injection method

After anesthesia satisfaction, 1 mL of CNSI (Chongqing Lummy Pharmaceutical Co., Ltd., Chongqing, China) was taken, and the inner or outer plate of the foreskin was selected at approximately 0.5–1.0 cm around the penile mass for injection. Attention was given to avoiding the tumor. The injection site can be adjusted appropriately around the tumor.

### Statistical methods

SPSS 16.0 statistical software was used for analysis. Measurement data are expressed as the mean ± standard deviation (x ± s). Mann–Whitney *U*-test and a Chi-square test were used, and *p* < 0.05 was considered statistically significant.

## Results

### CNSI as a sentinel lymph node tracer for penile cancer has high accuracy and safety and does not additionally influence the postoperative appearance and pathological analysis

**Figure 1:**
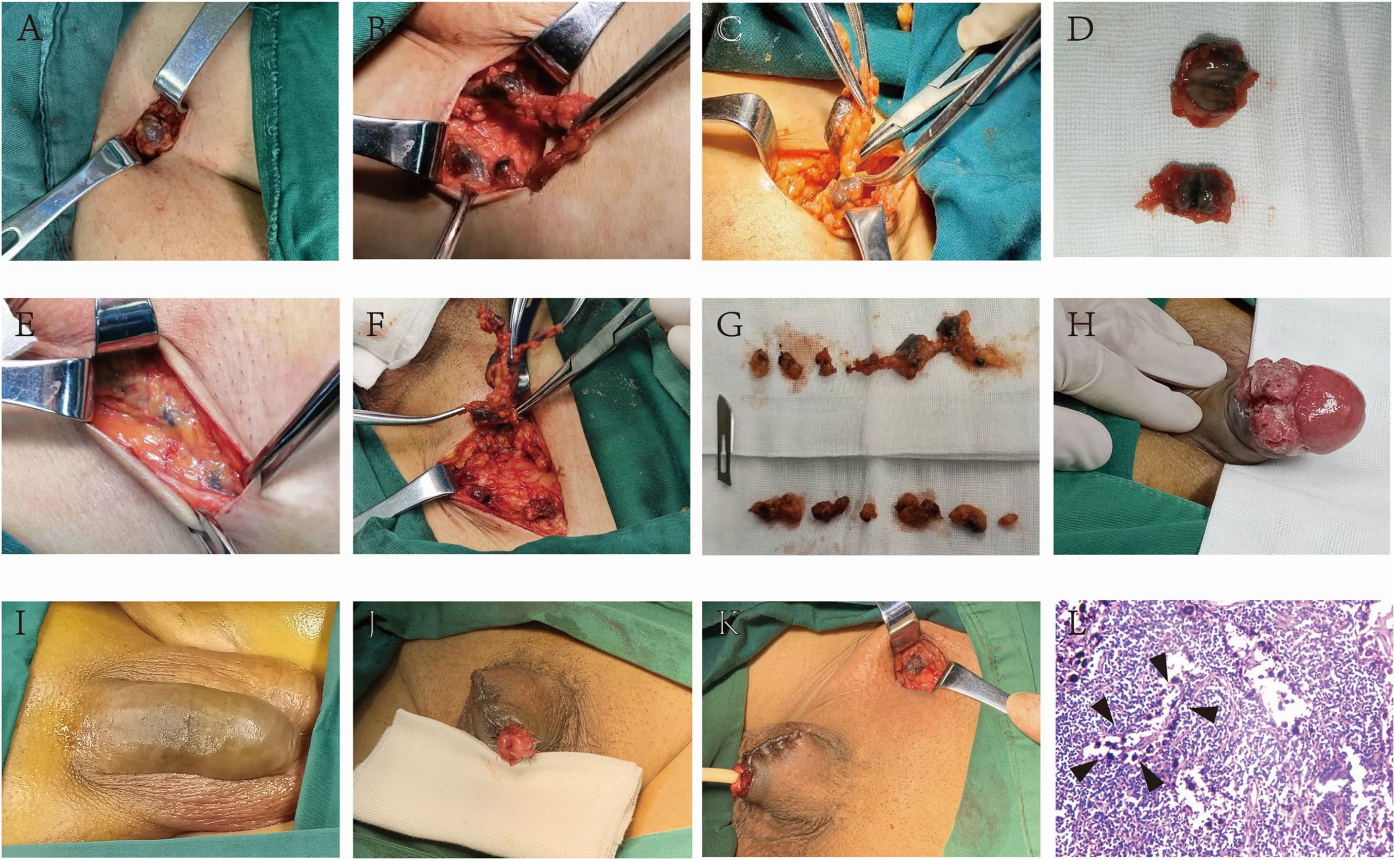
CNSI as a sentinel lymph node tracer for penile cancer has high accuracy and does not additionally influence the postoperative appearance and pathological analysis. The lymph node surface and cross-section showed a black staining appearance after the CNSI was applied **(A–G)**. The black-stained area caused by the injection of CNSI **(H,I)** is generally excised with the penis without additionally affecting the postoperative appearance **(J,K)**. HE staining of lymph node specimens showed that the CNSI was located at the edge of the lymphatic sinus and did not interfere with the reading of pathological sections **(L)**.

CNSI is effective as a tracer of inguinal sentinel lymph nodes in penile carcinoma. The lymph node surface (Figures 1A–C) and cross-section (Figure 1D) showed a black staining appearance. Sometimes, black-stained lymph nodes can be found when dissociating to Camper’s fascia (Figure 1E), and even more than 10 lymph nodes can be found on one side (Figures 1F,G). No auxiliary equipment is needed to achieve naked-eye lymphatic tracing; the black-stained area caused by the injection of CNSI (Figures 1H,I) is generally excised with the penis without additionally affecting the postoperative appearance (Figures 1J,K); H&E staining of lymph node specimens showed that the CNSI was located at the edge of the lymphatic sinus and did not interfere with the reading of pathological sections (Figure 1L). No obvious adverse reactions including fever, fatigue, allergy, and local redness, etc. or surgical complications occurred in any patients who received CNSI injections.

### CNSI helps to detect sentinel lymph nodes and improve surgical efficiency

The detection rate (DR) of lymph node, the number of lymph nodes sent for each case (NOLNSFEC), positive rate of lymphatic metastasis (PROLM) and operation time (OT) of each group were statistically analyzed, and the results were shown in Table 2. Among the 17 patients in the control group, 14 patients were successfully detected with at least one sentinel lymph node bilaterally. A total of 51 lymphatic pathological tissues were sent for examination, and 48 lymph nodes were detected. The remaining 3 patients were confirmed to have adipose tissue. The DR of lymph nodes was 94.12% (48/51). On average, the NOLNSFEC was 2.82 ± 0.78; the PROLM was 11.76% (2/17); and the OT was 42.41 ± 8.73 min. During follow-up, 1 out of 15 negative patients was found to have unilateral lymph node enlargement, which was confirmed as positive lymph node metastasis by puncture pathology. Therefore, the false negative rate in the control group was 33.3% (1/3).

**Table 2:** Data of different groups and statistical analysis results.

Project	Group		*p-*value	Group		*P*-value
	Control group	12HBS group		0.5HBS group	12HBS group	
NOC	17	6	/	6	6	/
DR	94.12%(48/51)	100.0%(72/72)	*p* = 0.04	100.0%(27/27)	100.0%(72/72)	/
			χ^2^ = 4.34			
NOLNSFEC	2.82 ± 0.78	12.0 ± 6.09	*p* = 0.001	4.50 ± 1.44	12.0 ± 6.09	*p* = 0.09
PROLM	11.76%(2/17)	16.67%(1/6)	*p* = 0.76	0%(0/6)	16.67%(1/6)	*p* = 0.29
			χ^2^ = 12.86			χ^2^ = 1.09
OT(min)	42.41 ± 8.73	43.67 ± 15.78	0.66	24.5 ± 5.89	43.67 ± 15.78	0.02

Among the 6 patients in the 12HBS group, at least 1 sentinel lymph node was successfully detected bilaterally in each patient. A total of 72 lymphatic pathological tissues were sent for inspection, and 72 lymph nodes were detected with a DR of 100% (72/72). An average of 12.0 ± 6.09 lymph nodes were sent for each patient; the PROLM was 16.67% (1/6); and the OT was 43.67 ± 15.78 min. Although there was no significant difference in the PROLM and the OT between the two groups, compared with the control group, the DR of lymph nodes in the 12HBS group was higher, and more lymph nodes were removed and sent for inspection (*p* < 0.05), which improved the efficiency of surgery.

Among the 6 patients in the 0.5HBS group, at least 1 sentinel lymph node was successfully detected bilaterally in each patient. A total of 27 lymph node pathological tissues were sent for examination, and 27 lymph nodes were detected with a DR of 100% (27/27). The NOLNSFEC was 4.50 ± 1.44; the PROLM was 0% (0/6); and the OT was 24.5 ± 5.89 min. Compared with the 12HBS group, the number of lymph nodes removed in the 0.5HBS group was reduced; the OT was shortened; and there was no significant difference in the DR and PROLM. In addition, in the 12HBS group and 0.5HBS group, no evidence of lymph node positive metastasis has been found in patients with penile cancer with negative sentinel lymph nodes. Therefore, we believe that the false negative rate in these two groups is 0%(0/1).

## Discussion

Lymph node metastasis is the main method by which penile cancer spreads. The number and extent of positive lymph nodes are related to the patient’s survival prognosis and treatment strategy. Therefore, preoperative and intraoperative lymph node localization and tracing are particularly important.

The shape, texture, and color of lymph nodes are similar to those of adipose tissue. It is often difficult to identify lymph nodes in adipose tissue, or fat, during surgery. Lymphatic tracers can help surgeons distinguish lymph nodes from ordinary adipose tissue and identify sentinel lymph nodes during surgery. There are a variety of lymphatic tracers. The CNKI used in this project exists in the form of aggregates with an average particle size of 50~300 nm, and each aggregate is composed of carbon nanoparticles with a particle size of about 10~50 nm (12). The capillary endothelial cell gap was 20~50 nm; the lymphatic capillary endothelial cell gap was 120–500 nm; and the basement membrane was not fully developed; thus, when the CNSI was injected into the local tissue, due to the pressure difference between the tissue fluid and the lymph fluid, the centripetal flow of the lymph fluid, as well as macrophages, can pass through the lymphatic capillaries. Alternatively, incomplete basement membrane and endothelial cell space quickly enter the lymphatic vessels (13) through infiltration and diffusion and stay and accumulate in the lymph nodes wherein they were stained black for easy identification and removal during surgery. Currently, increasing evidence shows that the application of CNSI in the lymphatic tracing of gastric cancer (14), colorectal cancer (15), breast cancer (16, 17), and thyroid cancer (18) has a definite effect. The effect is accurate as it guides the surgeon in delineating the scope of lymph node dissection while avoiding unnecessary trauma caused by enlarged lymph node dissection; it further aids the removal of additional lymph nodes during the surgery thereby providing more accurate pathological staging and guiding clinicians to formulate further standards. The follow-up treatment plan is to strive for survival benefits for the patient. We applied CNSI to patients with penile cancer (≥pT1G2 and cN0) in the tracing of sentinel lymph nodes in the groin. The results showed that (1) CNSI was effective as a sentinel lymph node tracer for penile cancer, and the bilateral inguinal sentinel lymph nodes were stained. Even when dissociated to Camper’s fascia, black-stained lymph nodes could be found, which allowed the operator to easily find and remove the lymph nodes. It is worth mentioning that the tracer realizes naked-eye tracing of lymph nodes without auxiliary equipment. This simplifies the surgical procedure, and costs are saved. (2) Most of the black-stained areas caused by CNSI injection were removed with penile excision, which does not additionally affect the postoperative appearance, which means that the use of activated carbon will not increase the additional, psychological burden on patients. (3) The CNSI tracer does not affect the pathological analysis and may help the pathological convergence. (4) Compared with the control group, the DR of lymph nodes in the group in which CNSI was injected 12 h before surgery was higher, and the number of lymph nodes sent for inspection was even more than 10 on one side. We believe that this demonstrates that, on the one hand, due to the tracer of CNSI, it avoids the occurrence of traditional surgeons mistaking adipose tissue for lymph nodes for examination. On the other hand, CNSI has extremely high sensitivity as a tracer, and the possibility of tracer failure is small, which is beneficial to reducing the risk of missing “micrometastases.” (5) We found that there was no significant difference in the PROLM between the control group and 12HBS group. The possible reason is similar to other tracers; CNSI cannot identify positive lymph nodes, which may also be related to the small number of enrolled cases. (6) The statistical analysis concluded that there was no significant difference in the OT between the control group and the 12HBS group. However, we believe that in the same OT, the 12HBS group removed more lymph nodes, which helped to improve the efficiency of the operation. (7) No obvious adverse reactions or surgical complications occurred in any of the patients injected with CNSI, which shows that CNSI is highly safe.

The sentinel lymph node is not necessarily located in a specific anatomical area, especially for patients with penile cancer whose inguinal lymph nodes cannot be palpated in clinical practice. It is often difficult to identify the lymph nodes during surgery, and it is even more difficult to identify which one is the sentinel lymph node. It can be seen that the use of CNSI tracing technology has great significance. However, among the many black-stained lymph nodes, which is the “most anterior position” sentinel lymph node is worth in-depth investigation. Does the increased number of lymph nodes removed increase unnecessary trauma? Based on these two considerations, we shortened the preoperative injection time of CNSI and changed it from 12 h before surgery to 0.5 h before surgery. The results showed that the number of lymph nodes sent for inspection is reduced in the 0.5HBS group, which did not affect the DR of lymph nodes, shortened the surgery time, and reduced surgical trauma. In addition, This narrows down the “most anterior position” sentinel lymph node. Regarding the timing of preoperative injection of CNSI, we will further accumulate more cases and finally summarize the best preoperative injection time.

We believe that the most important operation step of CNSI tracing technology is the injection site of CNSI because this involves whether the lymph nodes sent for examination are “sentinel lymph nodes”; otherwise, it may cause “false negative” results leading to “invalid tracing.” Here, we share some of our experiences of injecting CNSI: (1) It must be injected around the tumor; in this way, the black-stained inguinal lymph nodes are the sentinel lymph nodes of our target. (2) Inject as far as possible into the inner plate of the foreskin because there are abundant lymphatic pathways here, and it is relatively easier to grasp the depth of injection. For patients with a large area of the inner plate invaded by the tumor, it can also be injected through the outer plate of the foreskin. (3) The oblique puncture needle should not be too deep, and the sneak distance should be as long as possible. Before injection, it should be withdrawn to confirm that it has not entered the blood vessel by mistake. The injection speed should be as slow as possible. After the injection, the syringe should be withdrawn to form a “negative pressure state” so as to close the needle path and reduce injection spillage. (4) After injection, the gauze was quickly pressed for 3–5 min and properly pressed, wrapped, and kneaded to promote lymphatic reflux. (5) Do not inject into the tumor body because the lymphatic pathway in the tumor was destroyed, and the ideal tracing effect cannot be obtained.

## Conclusion

In conclusion, the application of CNSI for naked-eye tracing of inguinal sentinel lymph nodes in penile cancer is safe and efficient. There was no need for auxiliary equipment, almost no additional impact on postoperative appearance, and no impact on pathological analysis. Injecting CNSI 0.5 h before surgery can help to identify the “most anterior position” sentinel lymph nodes and reduce surgical trauma.

## Data availability statement

The original contributions presented in the study are included in the article/supplementary material, further inquiries can be directed to the corresponding author.

## Ethics statement

The studies involving human participants were reviewed and approved by the Medical Ethics Committee of LU’AN Affiliated Hospital of Anhui Medical University. The patients/participants provided their written informed consent to participate in this study. Written informed consent was obtained from the individual (s) for the publication of any potentially identifiable images or data included in this article.

## Author contributions

CLiu designed all the research projects, analyzed data, and wrote the manuscript. PX and MY revised and proofread the manuscript. CLiu, PX, SS, MY, CLi, SL, WL, XD, JM, and GL participated in the successful completion of the surgery. All authors contributed to the article and approved the submitted version.

## Funding

This study was supported by Health Research Project of Anhui Province in 2022 (nos. AHWJ2022c010 and AHWJ2022a033).

## Conflict of interest

The authors declare that the research was conducted in the absence of any commercial or financial relationships that could be construed as a potential conflict of interest.

## Publisher’s note

All claims expressed in this article are solely those of the authors and do not necessarily represent those of their affiliated organizations, or those of the publisher, the editors and the reviewers. Any product that may be evaluated in this article, or claim that may be made by its manufacturer, is not guaranteed or endorsed by the publisher.

## Abbreviations

12HBS group: The group in which CNSI was injected 12 h before the surgery
0.5HBS group: The group in which CNSI was injected 0.5 h before the surgery
DR: The detection rate
NOLNSFEC: The number of lymph nodes sent for each case
PROLM: Positive rate of lymphatic metastasis
OT: Operation time
